# Supplementary material for: Reconciling Mining with the Conservation of Cave Biodiversity: A Quantitative Baseline to Help Establish Conservation Priorities
Source: PLoS One. 2016 Dec 20;11(12):e0168348. doi: 10.1371/journal.pone.0168348 (PMC5173368; doi:10.1371/journal.pone.0168348)
Supplement: S1 Dataset — (ZIP) [file pone.0168348.s002.zip › Taxa/Serra Norte/SN_2013/N4E/Lista N4E-85.pdf]

CAVIDADE N4E-0085

Classe	Ordem	Fam/Outros	Gên/Outros	Espécie	S-U	U-S	Zon.
Arachnida	Acari	(Oribatida)		sp.3		X	A
Arachnida	Acari	Eupodidae		sp.1	X		A
Arachnida	Acari	Gamasida		jovem		X	A
Arachnida	Acari	Ixodidae	*Amblyomma*	sp.1		X	A
Arachnida	Acari	Laelapidae	*Cosmolaelaps*	sp.1		X	P
Arachnida	Acari	Laelapidae		sp.1		X	A
Arachnida	Acari	Laelapidae		sp.3	X	X	P/A
Arachnida	Acari	Macronyssidae		sp.1		X	A
Arachnida	Acari	Mesostigmata		jovem	X		E/P/A
Arachnida	Acari	Neothyridae	*Diplothyrus*	jovem		X	P/A
Arachnida	Acari	Podocinidae	*Podocinum*	sp.1	X		E
Arachnida	Acari	Veigaiidae		jovem		X	A
Arachnida	Amblypygi	Charinidae	*Charinus*	sp.1	2	1	A
Arachnida	Amblypygi	Phrynidae	*Heterophrynus*	*longicornis*	1	1	A
Arachnida	Araneae	Barychelidae		jovem		1	E
Arachnida	Araneae	Corinnidae		jovem		1	A
Arachnida	Araneae	Ctenidae		jovem	1	2	E/A
Arachnida	Araneae	Ochyroceratidae	*Ochyrocera*	sp.3	X	X	A
Arachnida	Araneae	Paratropididae		jovem		1	E
Arachnida	Araneae	Pholcidae	*Mesabolivar*	sp.1	x	X	E
Arachnida	Araneae	Segestriidae		jovem	X	X	E/A
Arachnida	Araneae	Theridiosomatidae	*Plato*	sp.1	X	X	P/A
Arachnida	Opiliones	Cosmetidae	*Roquettea*	*singularis*	1		E
Arachnida	Opiliones	Escadabiidae		sp.2	X		A
Arachnida	Pseudoscorpiones	Bochicidae		sp.1	X	X	A
Arachnida	Pseudoscorpiones	Chthoniidae	*Pseudochthonius*	sp.1	X	X	E/A
Arachnida	Schizomida	Hubbardiidae	aff. *Rowlandius*	sp.1	X	X	A
Chilopoda	Scolopendromorpha	Scolopocryptopidae	*Dinocryptops*	*miersii*		1	A
Chilopoda	Scolopendromorpha	Scolopocryptopidae	*Tidops*	sp.1		2	A
Diplopoda	Polydesmida	Chelodesmidae		sp.3	1	13	P/A
Diplopoda	Polydesmida	Fuhrmannodesmidae		sp.1	X	X	P/A
Entognatha	Collembola	Cyphoderidae		sp.1	X	X	E/P
Entognatha	Collembola	Isotomidae		sp.1	X	X	E/P/A
Entognatha	Collembola	Paronellidae		sp.4	X	X	E/P/A
Entognatha	Collembola	Sminthuroidea		sp.2	X	X	P/A
Entognatha	Diplura	Campodeidae		sp.1	X	X	E/A
Entognatha	Diplura	Japygidae		sp.1	X	X	P/A
Insecta	Blattodea	Blattellidae		sp.1	1	5	A
Insecta	Coleoptera	Carabidae		sp.3	X	X	E/P/A
Insecta	Coleoptera	Dytiscidae		sp.16		X	A
Insecta	Coleoptera	Scydmaenidae		sp.1		X	A
Insecta	Coleoptera	Scydmaenidae		sp.2	X		A
Insecta	Coleoptera	Scydmaenidae		sp.3	X	X	A
Insecta	Coleoptera	Scydmaenidae		sp.5		X	A
Insecta	Coleoptera	Staphylinidae	Pselaphinae	sp.32	X		A
Insecta	Coleoptera	Staphylinidae	Pselaphinae	sp.35		X	A
Insecta	Coleoptera	Staphylinidae		sp.65		X	P
Insecta	Diptera	Cecidomyiidae	Cecidomyiinae	sp.		X	E
Insecta	Diptera	Chironomidae		sp.		X	A

Insecta	Diptera	Culicidae	*Culex*	sp.	X	X	E/A
Insecta	Diptera	Psychodidae	Phlebotominae	sp.	X	X	E/P/A
Insecta	Diptera	Sciaridae		sp.		X	A
Insecta	Diptera	Tipulidae	Tipulinae	sp.	X	X	E/P
Insecta	Heteroptera	Cydnidae		jovem		X	P
Insecta	Homoptera	Cixiidae		jovem	X	X	E/P/A
Insecta	Hymenoptera	Diapriidae		sp.1		X	A
Insecta	Hymenoptera	Formicidae	*Camponotus*	sp.2		X	P
Insecta	Hymenoptera	Formicidae	*Pachycondyla*	sp.1	X	X	E
Insecta	Hymenoptera	Formicidae	*Pachycondyla*	sp.2	X	X	E/P/A
Insecta	Hymenoptera	Formicidae	*Paratrechina*	sp.2		X	E
Insecta	Hymenoptera	Formicidae	*Pheidole*	sp.2	X		E
Insecta	Isoptera	Termitidae	*Nasutitermes*	sp.	X	X	E/P/A
Insecta	Lepidoptera	Noctuoidea		sp.5	1	5	E/P
Insecta	Orthoptera	Phalangopsidae	*Paraclodes*	jovem	1	2	E
Insecta	Orthoptera	Phalangopsidae	*Phalangopsis*	sp.	4	2	A
Insecta	Psocoptera	Psyllipsocidae	*Psyllipsocus*	sp.1		X	A
Insecta	Thysanura	Nicoletiidae		sp.1	X	X	P/A
Gastropoda	Pulmonata	Bulimulidae	*Naesiotus*	sp.	X	X	E/P/A
Gastropoda	Pulmonata	Subulinidae		sp.4	X	X	E/P/A
Gastropoda	Pulmonata	Systrophiidae	*Happia*	sp.	X	X	E/A
Malacostraca	Isopoda	Armadillidae		sp.1		X	A
Malacostraca	Isopoda	Dubioniscidae		sp.1		X	E
Malacostraca	Isopoda	Scleropactidae	*Circoniscus*	*buckupi*	X		E/A
Turbellaria	Tricladida			sp.4		2	P
Turbellaria	Tricladida			sp.5		6	A
Amphibia	Anura	Strabomantidae	*Pristimantis*	cf. *fenestratus*	11		P
Mammalia	Chiroptera	Emballonuridae	*Peropteryx*	*kappleri*	5	1	E/P
Mammalia	Chiroptera	Furipteridae	*Furipterus*	*horrens*		2	P
Mammalia	Chiroptera	Phyllostomidae	*Carollia*	*perspicillata*		2	P
Mammalia	Chiroptera	Phyllostomidae	*Diphylla*	*ecaudata*	1	1	A
